# Purchase Intention towards Alternative Medicine: A Study from Consumers’ Perspective in Malaysia

**Published:** 2020-01

**Authors:** Ahasanul HAQUE, Naila ANWAR, Farzana YASMIN, Arun KUMAR TAROFDER, Nazmul Mohammad Hassan MAZIZ

**Affiliations:** 1 Department of Business Administration, International Islamic University Malaysia, Kuala Lumpur, Malaysia; 2 Faculty of Science, Lincoln University, Jalan Stadium, Kelana Jaya, Malaysia; 3 Faculty of Business Management and Professional Studies, Management and Science University, Selangor, Malaysia Section 13, 40100 Shah Alam, Selangor Darul Ehsan, Malaysia; 4 Graduate School of Medicine, Perdana University, Jalan MAEPS, Perdana, Malaysia

## Dear Editor-in-Chief

**Fig. 1:**
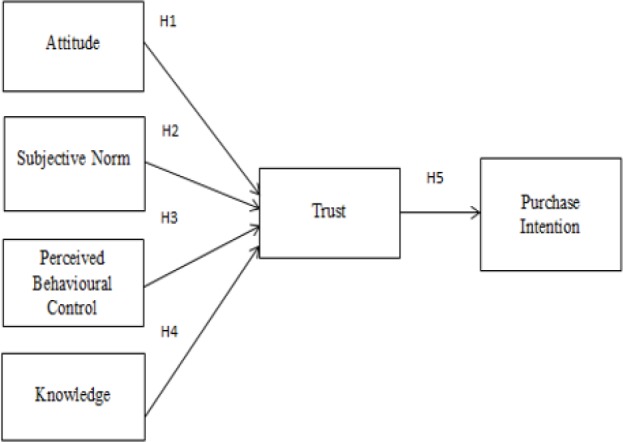
Conceptual Framework

Modern medication has proven to be effective in preventing and curing many types of illnesses and prolonging life of many people. However, as modern medicines rely on the issuance of synthetic medications, they produce negative side effects that practically supersedes the advantages that one gains from using them. Therefore, consumers are in search of other types of medications particularly, alternative medicines. Individuals’ awareness of living a healthy lifestyle is the primary reason that has increased the worldwide demand for alternative medicines (1). Alternative medicines are growing in popularity and consumers are seen to demonstrate a positive inclination toward purchasing but, a review of prior studies revealed that a comprehensive study on the aspects that may drive purchase intention of alternative medicines has not been conducted yet. For such reason, the current study has been developed with the ultimate objective of examining the relationship between attitude, subjective norm, perceived behavioural control, knowledge, trust, and purchase intention of alternative medicine, particularly in the setting of Malaysia. To fulfill the objective of the study, a conceptual framework has been developed as depicted in Fig. 1.

By observing the conceptual framework, it can see the study consists of five hypotheses:

H1: There is a positive relationship between attitude and trust in purchase intention of alternative medicine.
H2: There is a positive relationship between subjective norm and trust in purchase intention of alternative medicine.
H3: There is a positive relationship between perceived behavioural control and trust in purchase intention of alternative medicine.
H4: There is a positive relationship between knowledge and trust in purchase intention of alternative medicine.
H5: There is a positive relationship between trust and purchase intention of alternative medicine.

**Fig. 2:**
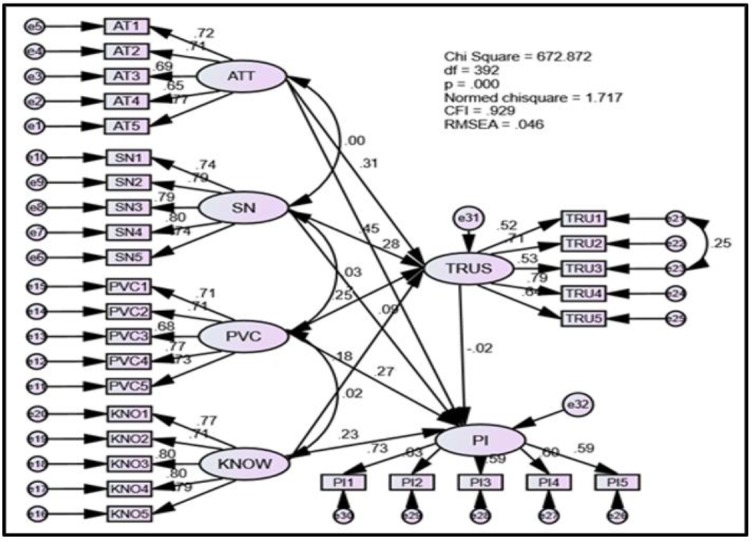
Structural Model

As the study was quantitative in nature, survey method was used to collect data through the means of self-administered questionnaires based on simple random sampling (2, 3). Data were gathered from Klang Valley, Kuala Lumpur, Malaysia with 350 respondents in 2018, who were more experienced in using alternative health products. Factor Analysis (EFA) was conducted at the initial stage and six respective factors were extracted. Moreover, all items under each of the constructs managed to load above the threshold value of .50 (3). The final stage, the researchers proceeded with the verification of the structural model to attest to the fitness of the full-fledged model and verify the hypotheses. Based on the Goodness of Fit (GOF) values obtained, the model can be considered fit as the requirements for each of the fitness indices were met as indicated in Fig. 2.

The results of the hypotheses testing, individuals’ attitude, subjective norm, perceived behavioral control along with knowledge are significant aspects that give rise to a feeling of trust. Such findings are supported by prior literature (4–7). However, in this study trust did not share a favourable affiliation with purchase intention and similar outcomes were generated in the past as well (8). Table 1 summarizes the results of hypotheses testing.

**Table 1:** Hypothesis Testing

***Hypotheses***	***Standardized Regression Weights***			***Estimate***	***S.E.***	***C.R.***	**P**	***Outcome***
H1	TRUST	<--	ATT	.267	.046	5.821	***	Supported
H2	TRUST	<--	SN	.206	.035	3.158	***	Supported
H3	TRUST	<--	PVC	.036	.087	3.972	.002	Supported
H4	TRUST	<--	KNOW	.096	.033	2.887	.004	Supported
H5	PI	<--	TRUST	.048	.081	.794	.331	Not Supported

Additionally, the mediating effect of trust was also verified. All the independent variables (IVs) share a significant, direct relationship with purchase intention (DV). Besides, all the IVs stated above also have a significant relationship with trust (MV) (Fig. 2).

However, the relationship between trust (MV) and purchase intention (DV) is not significant. Hence, trust does not mediate the relationship between the IVs and DV.

Based on the statistical outcomes, marketers should strive to create a positive attitude toward alternative medicine for building consumers’ trust. Moreover, when it comes to winning consumers’ trust and persuading them to purchase alternative medicine, perceived behavioral control plays a significant role. Thus, alternative medicine products are sold at an affordable price. It is necessary to work toward ameliorating social awareness and acceptance of alternative medicine and provide adequate information and knowledge about alternative medicine through various marketing strategies and promotional activities.
